# Dielectric-induced surface wave radiation loss

**DOI:** 10.1098/rspa.2019.0859

**Published:** 2020-04-08

**Authors:** Tobias Schaich, Anas Al Rawi, Trevor Morsman, Mike Payne

**Affiliations:** 1 Department of Physics - Cavendish Laboratory, University of Cambridge, Cambridge CB3 0HE, UK; 2 BT Labs, Adastral Park, Orion Building, Martlesham Heath, Ipswich IP5 3RE, UK

**Keywords:** surface waves, dielectric loss, transmission line, radiation

## Abstract

We investigate a model which shows how the introduction of a perturbing dielectric close to an electromagnetic surface wave leads to radiation away from the surface through the dielectric. This resembles a surface waveguide passing through a wall or being deployed underground. Our theory, which is based on the mode-matching technique, allows quantitative determination of losses from a bound surface wave mode up to the point of its complete extinction. For a surface wave supported by a coated, conducting sheet the attenuation due to the perturbing dielectric is calculated for a number of frequencies, permittivities of the perturbation and separations between the sheet and the perturbing dielectric. The accuracy of our results is verified by simulation of the system with a full-wave numerical solution. Finally, we report experimental data of perturbed surface waves on a cable, which are in qualitative agreement with our model.

## 1. Introduction

Electromagnetic surface waves in open waveguide structures are a means of transmitting signals in the GHz and THz range with low loss and dispersion compared with other closed waveguide structures [1–3]. Surface waveguides can, for instance, be single wires with finite conductivity [4] or perfectly conducting wires that have a dielectric sheath or are corrugated [5]. However, because these systems are open, the surface waves carried on them are susceptible to disturbances by the surrounding environment.

A number of previous works have studied the effect of different coatings and media surrounding a coated wire or related systems [6,7]. John and Chatterjee [8] reported that if the dielectric constant of the surrounding medium is higher than the dielectric constant of the coating the surface wave solution ceases to exist. However, if we imagine that the dielectric medium is moved very far away from the conductor and the opening gap is filled with vacuum or air, we would expect the surface wave to propagate again.

The question of how a neighbouring dielectric affects a propagating surface wave at intermediate distances between the dielectric and the surface is investigated in this paper. An approach based on the mode-matching technique [9] is developed and employed. To simplify the calculations, a one-dimensional system, i.e. an infinite, perfectly conducting plane coated with a lossless dielectric as discussed by Attwood [10], is considered as an example. However, qualitatively similar results would be expected for a cylindrical wire and other geometries. This is supported by experimental data using a cylindrical cable at varying distances from the ground. Hence, the conclusions of this paper can be used to model and understand the behaviour of a surface waveguide passing through a wall or being used underground.

## 2. Theoretical model

We first recapitulate the mode-matching method and state the approximations used in our theory. Consider a general system of two dissimilar waveguides (1 and 2) on either side of the *z* = 0 plane separated by a discontinuity. In this system the *z*-axis is the axis of wave propagation. The transverse components of any electric and magnetic field on either side of the discontinuity can be expanded in terms of incoming and outgoing eigenmodes of the relevant waveguide in the form [9]

2.1 $$E_{t}^{\left(i\right)}=\sum_{m}a_{m}^{\left(i\right)}e_{m}^{\left(i\right)}\exp(-j\beta_{m}z)+b_{m}^{\left(i\right)}e_{m}^{\left(i\right)}\exp(j\beta_{m}z)$$

and

2.2 $$H_{t}^{\left(i\right)}=\sum_{m}a_{m}^{\left(i\right)}h_{m}^{\left(i\right)}\exp(-j\beta_{m}z)-b_{m}^{\left(i\right)}h_{m}^{\left(i\right)}\exp(j\beta_{m}z).$$

Here, *i* denotes waveguide 1 or 2, *j* is the imaginary unit, $a_{m}^{\left(i\right)}$ and $b_{m}^{\left(i\right)}$ are the incoming and outgoing amplitudes to the mode *m* with propagation constant *β*_*m*_ and electric and magnetic fields $e_{m}^{\left(i\right)}$ and $h_{m}^{\left(i\right)}$, respectively. The sum is understood as summation over all bound modes and integration over the radiation and evanescent modes. The fields are assumed to oscillate at an angular frequency *ω* in time *t*. We will omit the common factor exp (*jωt*) describing the time evolution of the fields in the following.

At the discontinuity at *z* = 0, the transverse fields must be continuous

2.3 $$E_{t}^{\left(1\right)}{|}_{z=0}=E_{t}^{\left(2\right)}{|}_{z=0}$$

and

2.4 $$H_{t}^{\left(1\right)}{|}_{z=0}=H_{t}^{\left(2\right)}{|}_{z=0}.$$

Let ${\;}^{*}$ denote complex conjugation. Then, integrating the vector product of equation (2.3) with $h_{n}^{(2)*}$ and of $e_{n}^{(2)*}$ with equation (2.4) over the entire waveguide cross section at *z* = 0 yields

2.5 $$\sum_{m}2P_{mn}^{\left(2\right)}(a_{m}^{\left(2\right)}+b_{m}^{\left(2\right)})=2\sum_{m}(a_{m}^{\left(1\right)}+b_{m}^{\left(1\right)})I_{mn}$$

and

2.6 $$\sum_{m}2P_{mn}^{(2)*}(a_{m}^{\left(2\right)}-b_{m}^{\left(2\right)})=2\sum_{m}(a_{m}^{\left(1\right)}-b_{m}^{\left(1\right)})J_{mn},$$

where we used the eigenmode expansion of the fields. The complex power $P_{mn}^{\left(i\right)}$ and the interaction integrals *I*_*mn*_ and *J*_*mn*_ are defined as follows:

2.7 $$\begin{array}{rl} & P_{mn}^{\left(i\right)}=\frac{1}{2}\int \;\text{d}S(e_{m}^{\left(i\right)}\times h_{n}^{(i)*})\hat{z}, \end{array}$$

2.8 $$\begin{array}{rl} & I_{mn}=\frac{1}{2}\int \;\text{d}S(e_{m}^{\left(1\right)}\times h_{n}^{(2)*})\hat{z} \end{array}$$

2.9 $$\begin{array}{rl} \;\text{and}\; & J_{mn}=\frac{1}{2}\int \;\text{d}S(e_{n}^{(2)*}\times h_{m}^{\left(1\right)})\hat{z}, \end{array}$$

where $\hat{z}$ is the unit vector in the *z*-direction and integration is over the entire cross-section of the waveguides.

Using the orthogonality of modes assuming no contribution of complex modes, $P_{mn}^{\left(i\right)}$ reduces to a constant multiplied by a Kronecker delta in the discrete and a delta function in the continuous case [11,12]. We may write this as

2.10 $$P_{mn}^{\left(i\right)}=\delta_{mn}P_{n}^{\left(i\right)},$$

with *δ*_*mn*_ being the Kronecker delta. Summation over *m* leaves us with $P_{n}^{\left(i\right)}$. Note that, as we are not taking the real part of *P*_*n*_, it is non-zero for evanescent modes. Assuming a single mode incident on the junction from *z* < 0 with no reflections further down the line, the matching conditions (2.5) and (2.6) take the form

2.11 $$P_{n}^{\left(2\right)}a_{n}^{\left(2\right)}=a_{1}^{\left(1\right)}I_{1n}+\sum_{m}b_{m}^{\left(1\right)}I_{mn}$$

and

2.12 $$P_{n}^{(2)*}a_{n}^{\left(2\right)}=a_{1}^{\left(1\right)}J_{1n}-\sum_{m}b_{m}^{\left(1\right)}J_{mn}.$$

This means that even for a single mode input we end up with an infinite set of equations as both *n* and *m* become continuous indices for the radiation modes. Thus, the matching problem is, in general, very difficult to solve. Therefore, we will use an approximation where both waveguides are assumed to be similar enough so that reflections can be considered small. Then, the transmitted power and excited modes are mainly dependent on the incoming wave rather than the reflected wave. This is equivalent to setting $b_{m}^{\left(1\right)}$ in equations (2.11) and (2.12) equal to zero. Clearly, this will lead to some ambiguity as we now have two equations for $a_{n}^{\left(2\right)}$, which in the general case will not give equal values. However, the difference between the two equations depends on the dissimilarity of the two waveguides and their eigenmodes. Thus, it indirectly scales with the magnitude of the reflection. Therefore, in the regime where our approximation is valid these two equations should give similar expansion coefficients.

**Figure 1.**
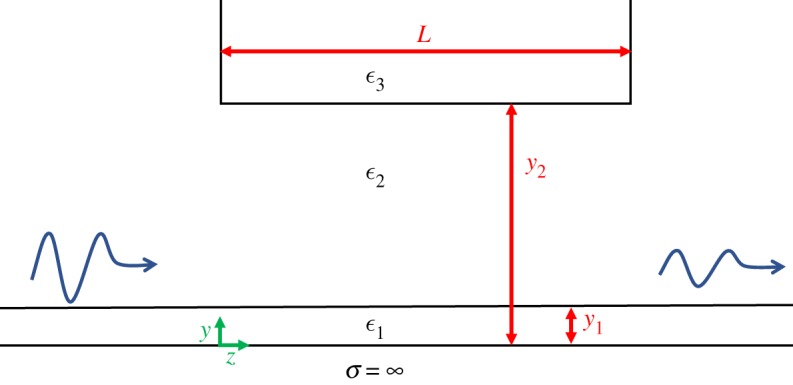
Schematic of the perturbed surface waveguide. A perfectly conducting plane, coated by a dielectric *ϵ*_1_ of thickness *y*_1_ and surrounded by a material with dielectric constant *ϵ*_2_, is perturbed by a dielectric *ϵ*_3_ at a distance *y*_2_ from the conductor. It extends for a length *L* along the propagation direction. The system is assumed to be infinite in the paper plane and a two-dimensional Cartesian coordinate system (*y*, *z*) is shown. A signal incident from the left is attenuated as part of the energy is turned into radiation over the perturbed region. (Online version in colour.)

In the next step, we apply the developed method to the one-dimensional surface waveguide described by Attwood: a perfectly conducting plane covered by a dielectric coating of thickness *y*_1_ and permittivity *ϵ*_1_ surrounded by a medium with dielectric constant *ϵ*_2_. This waveguide is perturbed over a length *L* by another dielectric with dielectric constant *ϵ*_3_ positioned at a distance *y*_2_ from the conductor. A sketch of the system can be seen in figure 1. We will assume that the unperturbed waveguide is operated at a frequency where only a single bound mode (denoted by subscript 1) can propagate and are interested in the amplitude of this mode beyond the perturbed region. Using the method described above at both discontinuities between the perturbed and unperturbed waveguide, we find that the amplitudes of the bound mode before and after the perturbation, $a_{1}^{\text{in}}$ and $a_{1}^{\text{out}}$, are related by

2.13 $$a_{1}^{\text{out}}\approx \sum_{m}a_{1}^{\text{in}}\frac{I_{1m}J_{1m}^{*}}{P_{1}^{\left(1\right)}P_{m}^{\left(2\right)}}\exp(-j\beta_{m}L).$$

It is easy to show that for the case *ϵ*_3_ = *ϵ*_2_, i.e. when there is no perturbation from the perfect waveguide, equation (2.13) gives $a_{1}^{\text{out}}=a_{1}^{\text{in}}\exp(-j\beta_{1}L)$, as we would expect for a single mode propagating a distance *L*. To calculate the transmitted amplitude for any other value of *ϵ*_3_ we will need to determine the full mode structure of the perturbed waveguide. This will be discussed in the next section.

## 3. Modes of the perturbed waveguide

The bound modes of the unperturbed guide have been discussed by Attwood [10]. However, if we introduce the perturbing dielectric with *ϵ*_3_ > *ϵ*_1_ there are no lossless, bound TM modes, as we show in appendix A. Thus, we only need to consider radiating and evanescent modes. Omitting the wave propagation factor of exp (*j*(*ωt* − *βz*)) these are given by the following fields:

3.1 $$\begin{array}{rl} E_{z} & =\left\{ \begin{array}{ll} E_{1}\sin(h_{1}y)\; & 0\leq y\leq y_{1},\\ E_{2}\sin(h_{2}y+\varphi ) & y_{1}\leq y\leq y_{2},\\ E_{3}\sin(h_{3}y+\psi ) & y_{2}\leq y, \end{array}\right. \end{array}$$

3.2 $$\begin{array}{rl} H_{x} & =j\omega \left\{ \begin{array}{ll} E_{1}\frac{\epsilon_{1}}{h_{1}}\cos(h_{1}y) & 0\leq y\leq y_{1},\\ E_{2}\frac{\epsilon_{2}}{h_{2}}\cos(h_{2}y+\varphi ) & y_{1}\leq y\leq y_{2},\\ E_{3}\frac{\epsilon_{3}}{h_{3}}\cos(h_{3}y+\psi ) & y_{2}\leq y, \end{array}\right. \end{array}$$

3.3 $$\begin{array}{rl} E_{y} & =-j\beta \left\{ \begin{array}{ll} E_{1}\frac{1}{h_{1}}\cos(h_{1}y) & 0\leq y\leq y_{1},\\ E_{2}\frac{1}{h_{2}}\cos(h_{2}y+\varphi ) & y_{1}\leq y\leq y_{2},\\ E_{3}\frac{1}{h_{3}}\cos(h_{3}y+\psi ) & y_{2}\leq y \end{array}\right. \end{array}$$

3.4 $$\begin{array}{rl} \text{and}\;h_{i} & =\sqrt{\omega^{2}\epsilon_{i}\mu_{0}-\beta^{2}}, \end{array}$$

where φ and *ψ* are possibly complex angles determined by the boundary conditions at *y*_1_ and *y*_2_ for a given *β*. The amplitudes *E*_1_, *E*_2_, *E*_3_ are related by the continuity of *E*_*z*_. The permeability of vacuum is denoted *μ*_0_. Modes exist both for real and for imaginary values of *β* corresponding to radiating and evanescent modes. A convenient way of labelling the modes is by their value of *h*_3_ as this quantity is real for all physical values of *β*. More explicitly, if *h*_3_ were imaginary the fields would diverge as *y* tends to infinity. So, from our choice of *h*_3_ we can calculate *β* and, consequently, *h*_1_ and *h*_2_ as well as φ and *ψ*. If *h*_3_ is less than $\omega \sqrt{\epsilon_{3}\mu_{0}}$ the modes are radiating away from the conductor. Otherwise, they are evanescent. The continuum of modes can be normalized to a Dirac delta function as follows:

3.5 $$\frac{1}{2}\int_{0}^{\infty }(E_{h_{3}}\times H_{h_{3}^{\prime }}^{*})\hat{z}\;\text{d}y=E_{3}E_{3}^{\prime *}\frac{\pi \beta \omega \epsilon_{3}}{4h_{3}h_{3}^{\prime *}}\delta (h_{3}-h_{3}^{\prime }),$$

where all primed quantities correspond to $H_{h_{3}^{\prime }}^{*}$.

In the case where *ϵ*_1_ > *ϵ*_3_ there can be a bound mode in addition to the radiation and evanescent modes described above. The existence of a bound mode depends on whether there is a solution to the characteristic equation (A 5) derived in appendix A. The fields are given there as well.

## 4. Results

With the mode structure described here and in [10] the interaction integrals in equations (2.8) and (2.9) can be calculated analytically. As we have shown, the modes of the perturbed guide are completely defined for a given value of *h*_3_. So, the output amplitude can be calculated by expressing the sum in equation (2.13) as an integral over *h*_3_ for the radiation and evanescent modes and by adding the contribution of any bound modes if they exist. Because of the increasingly fast decay of the evanescent modes in the *z*-direction with increasing *h*_3_, the integral can be truncated at a sufficiently large value of *h*_3_. Thus, we only need to calculate a finite number of modes and can then interpolate in between them to find the behaviour of our system. In our calculations we used of the order of $O(10^{5})$ to $O(10^{6})$ excited modes. By calculating the power contained in the bound surface wave mode after the perturbing dielectric, it can be established how strongly the perturbation affects the bound mode. This allows us to assess if the bound mode may be used for signal transmission in more realistic scenarios.

To obtain quantitative understanding of the energy losses in a real system, we consider the attenuation of a surface wave propagating along a waveguide consisting of a conductor coated with Teflon in air perturbed by glass. The dielectric constants of Teflon, air and glass relative to the vacuum permittivity *ϵ*_0_ are taken as *ϵ*_1_/*ϵ*_0_ = 2.1, *ϵ*_2_/*ϵ*_0_ = 1 and *ϵ*_3_/*ϵ*_0_ = 5.5, respectively. The conductor and the dielectrics are assumed to be lossless throughout. Surface wave frequencies in the GHz range were considered.

**Figure 2.**
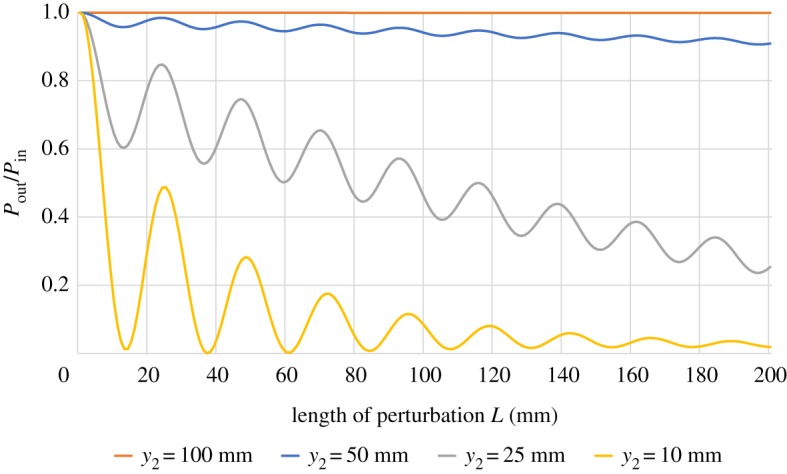
Transmitted power normalized to the input power as a function of the length of perturbation *L* for different distances *y*_2_ from the waveguide’s conductor (figure 1). The frequency of the input mode was kept at 10 GHz. Values of *ϵ*_1_/*ϵ*_0_ = 2.1, *ϵ*_2_/*ϵ*_0_ = 1, *ϵ*_3_/*ϵ*_0_ = 5.5 and *y*_1_ = 2 mm were used. The output power decreases with the length of perturbation. It can be seen that the transmitted power decreases more rapidly if the perturbing dielectric is closer. The oscillations are caused by interference effects between the excited modes. (Online version in colour.)

First, we investigate the effect of the perturbing dielectric at different distances *y*_2_ from the conductor at a fixed surface wave frequency of 10 GHz. The results are presented in figure 2. As we would expect, at very large distances of the perturbing dielectric from the conductor there is little to no attenuation of the surface wave. However, as the dielectric gets closer, energy is lost from the surface wave to radiation modes in the dielectric. This can lead to complete attenuation of the surface wave. We can think of the perturbing dielectric as opening a radiation channel allowing energy to propagate away from the surface. Hence, the surface wave loses energy. In addition to the average decrease of surface wave energy, we see a modulation on the output power with the length of the perturbation *L*. This can be attributed to the phase differences accrued by the multitude of excited modes while propagating along the perturbed waveguide section as each mode has a unique propagation constant *β*(*h*_3_). More specifically, the excited radiation modes have different phases *β*(*h*_3_) *L* due to propagation causing interference between these modes. This affects the amount of power that can return into the bound surface wave after the perturbation.

**Figure 3.**
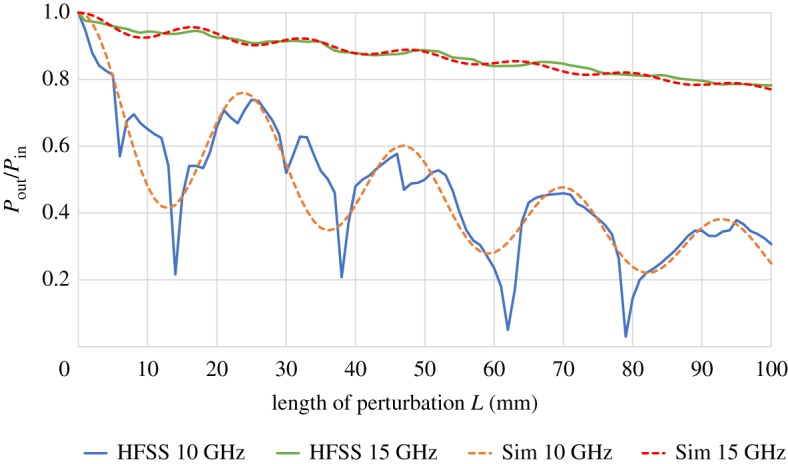
Transmitted power over input power as a function of the length of the perturbing dielectric *L*. The distance of the perturbation *y*_2_ was kept at 2 cm and the frequency was varied. Values of *ϵ*_1_/*ϵ*_0_ = 2.1, *ϵ*_2_/*ϵ*_0_ = 1, *ϵ*_3_/*ϵ*_0_ = 5.5 and *y*_1_ = 2 mm were used. Dotted lines are results derived using equation (2.13) while solid lines indicate results obtained through the finite element solver Ansys HFSS. (Online version in colour.)

In a next example, we keep the perturbation at a constant distance of *y*_2_ = 2 cm from the waveguide and vary the frequency of the incident surface wave. We expect a weak influence at high frequencies when the surface wave is tightly bound to the conductor and a stronger influence with decreasing frequency. This is exactly the behaviour predicted by our theory, as can be seen in figure 3. To validate our results and, hence, the accuracy of the approximations introduced to solve equations (2.5) and (2.6), we calculated the transmission characteristics of the same system with the finite element solver Ansys^®^ HFSS^TM^ [13]. Details of the HFSS model can be found at the end of this paper. We see good agreement between the simulations for high frequencies. As the frequency decreases, our approximation of negligible reflection becomes less valid and we observe stronger deviations between our results and those obtained using HFSS. In particular, the sharp peaks seen in the HFSS simulation are not found in our calculation.

**Figure 4.**
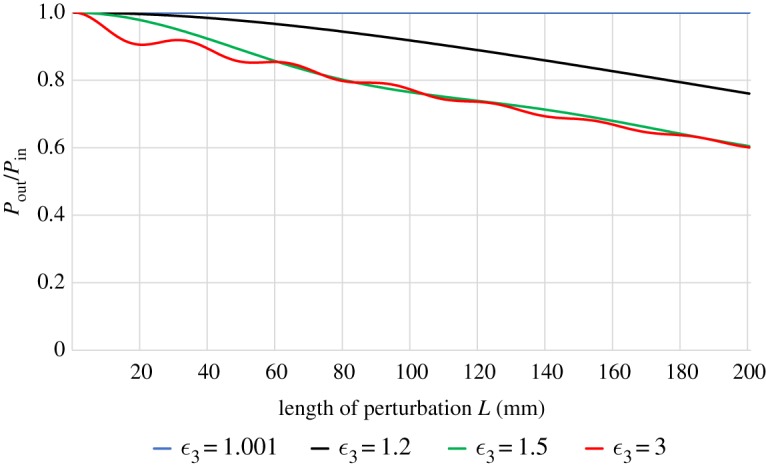
Normalized transmitted power as a function of the length of the perturbing dielectric *L*, which is a distance *y*_2_ = 2 cm away from the conductor at 15 GHz. The dielectric constant of the perturbation is varied and given in the legend as relative permittivities. When *ϵ*_3_/*ϵ*_2_ is approximately 1, no loss is seen. As it increases, the surface wave becomes more lossy until the effect saturates and the average loss becomes only weakly dependent on the permittivity except for very short perturbations. However, the interference pattern still depends on the exact dielectric constant. (Online version in colour.)

Finally, we look at the influence of the dielectric constant of the perturbing medium on the loss, leaving both the surface wave frequency and distance of the perturbation *y*_2_ constant at 15 GHz and 2 cm, respectively. Under these conditions, a bound mode in the perturbed system is possible if *ϵ*_3_/*ϵ*_0_ < 1.13. In figure 4, we show the transmitted power for different dielectric constants. The results indicate that at high dielectric constant *ϵ*_3_ the average loss of the surface wave depends only weakly on the exact value of the dielectric constant. Only the interference pattern and the loss for short perturbations is influenced. At lower values of the permittivity *ϵ*_3_, close to the onset of the bound mode and below, loss is reduced to zero as *ϵ*_3_ approaches the dielectric constant of the surrounding medium *ϵ*_2_.

**Figure 5.**
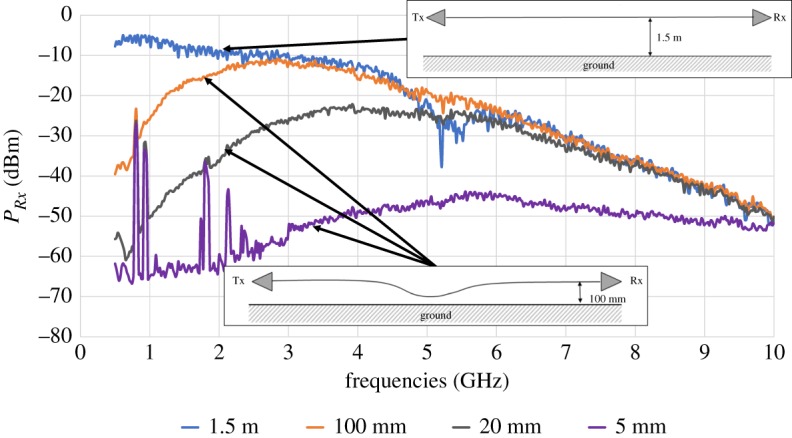
Experimental data of the received power through a 20 m cable suspended 100 mm above ground. The middle of the cable was brought to distances of 100 mm, 20 mm and 5 mm from the ground. A benchmark measurement with the entire cable suspended 1.5 m above the ground is included as well. The insets show schematics of the experimental set-up. When the cable is close to the ground high loss is observed. As the frequency increases behaviour similar to that of the benchmark measurement is recovered. (Online version in colour.)

To experimentally verify some predictions of this model, a 20 m long cable was suspended above ground at a height of 100 mm [14]. The cable was a Dropwire 11 with a single, 0.5 mm diameter annealed copper twisted pair with polyethylene insulation of thickness 0.215 mm for each conductor [15]. The twisted pair was contained in a polyethylene sheath of nominal diameter 5.3 mm and the steel strength members usually contained in the cable were removed. Surface waves were launched and received using horn launchers of 5 cm depth and 5 cm maximum cross-section. A signal generator was used as the source and a spectrum analyser as the receiver. The midpoint of the cable was brought close to the ground at distances of 100 mm (straight cable), 20 mm and 5 mm. As a benchmark the cable was also entirely suspended at 1.5 m above ground. The received power in these measurements is displayed in figure 5. The input power for all measurements was 0 dBm. As the horn launchers were not altered during the measurement, we believe their losses and launching efficiency to be constant throughout. The results in figure 5 show that at a given frequency the received power is reduced when the cable is brought close to the ground. Second, the results indicate that as the frequency increases the behaviour of a cable near the ground approaches the behaviour of a cable further from the ground. Both of these results are in qualitative agreement with the results presented in figures 2 and 3. Quantitative agreement cannot be established owing to the different geometries of the considered systems. Note that, because of the size of the experiment, measurements were carried out outside where the set-up was subjected to sources of radiation such as Wi-Fi, TV and cellular communications. As the observed spikes at low frequencies in figure 5 are at standard network carrier frequencies, we attribute them to our cable picking up some of this radiation.

## 5. Discussion and conclusion

As we have shown, surface waves are susceptible to disturbances of the surrounding medium. In fact, such disturbances can lead to the complete attenuation of a propagating wave. This occurs because of the perturbing dielectric opening a radiation channel into which the surface wave loses energy. Our model assumes negligible reflections, which should be accurate under the conditions that the perturbing dielectric is far away from the conductor. However, if the perturbation is close to the conductor, reflection can play an increasingly important role.

Quantifying *a priori* when reflections are small is not easy. One way to approach the problem is to consider the transverse decay constant $\kappa =\sqrt{\beta_{1}^{2}-\omega^{2}\epsilon_{2}\mu_{0}}$ of the unperturbed, bound mode, which describes how fast the fields decay away from the surface of the waveguide (*β*_1_ is the propagation constant of the bound mode [10]). In cases where *κy*_2_ ≫ 1 the fields have essentially decayed at the position of the perturbing dielectric and, so, there should not be any reflections. Even for values of *κy*_2_ ≈ 1 only about 13% of the power transmitted outside the coating is in the region *y* > *y*_2_. Thus, a sensible condition seems to be *κy*_2_ ≥ 1. In the examples seen in figure 3, which compares our model with a numerical solution, values of *κy*_2_ rounded to the second decimal place are 0.94 and 2.14 for 10 GHz and 15 GHz, respectively. Finally, it should be stressed that *κy*_2_ ≥ 1 is indicative of weak reflections but does not necessitate them.

Another important factor is mode-matching, meaning that both the perturbed and the unperturbed waveguide support modes with similar propagation constants. In our case the incident, unperturbed wave will have a propagation constant between $\omega \sqrt{\epsilon_{2}\mu_{0}}$ and $\omega \sqrt{\epsilon_{1}\mu_{0}}$. When *ϵ*_3_ > *ϵ*_1_ the perturbed waveguide always supports radiation modes in that region because all propagation constants below $\omega \sqrt{\epsilon_{3}\mu_{0}}$ belong to the radiating spectrum. However, in the case where *ϵ*_3_ < *ϵ*_1_ there is the possibility of poor matching. In particular, if *ϵ*_3_ is close to the threshold where the bound mode ceases to exist, a gap between the unperturbed propagation constant and the propagation constants of the perturbed bound and radiating modes is found to exist. Therefore, matching in this region of the dielectric constant *ϵ*_3_ might be poor, reflections can be strong and our model may produce inaccurate results.

As discussed, the developed model is valid under the condition of no or weak reflections. So, deviations from it are bound to increase as more power gets reflected at the discontinuities of the system. More specifically in the case considered above, the excited mode structure inside the perturbing dielectric region will deviate from the one calculated under the assumption of no reflections. Additionally, resonances which are due to re-reflections in the perturbing dielectric interfering constructively or destructively with the transmitted light are not included either. Within our approximation of negligible reflections their effect should be small. But, as reflections increase, they will play an important role for the exact description of the resulting transmission. This may explain the deviations seen for the 10 GHz wave in figure 3 between the presented model and numerical calculations.

Despite these restrictions, the measured data are in support of the presented model showing the predicted behaviour with frequency and distance from the perturbation. However, we acknowledge that losses may also increase simply because of increased dielectric loss inside the ground when the surface wave is brought into close proximity. Furthermore, the loss associated with the sagging of the line has not been considered separately. Thus, further measurements including radiation tests with different perturbing dielectrics are necessary for the verification of the model.

In summary, our results show a potential limitation of using surface waves for data transmission along cables. Specifically, use of the surface wave either underground or close to the ground seems impractical for large propagation distances unless the wave decays quickly enough in the transverse direction such that no perturbing medium nearby can influence it. On the one hand, our model shows that most dielectrics will cause some loss to the wave. On the other hand, it emphasizes that at increased frequencies these effects become weaker because the bound mode is more confined. Another way to enhance confinement is to increase the thickness of the dielectric coating. Therefore, careful design considerations need to be made when deploying surface waves as transmission lines.

In conclusion, we have derived a method to calculate the loss a perturbing dielectric induces on a bound surface wave by opening a radiation channel. The method is based on the approximation that reflections can be neglected and otherwise uses the well-established mode-matching technique. Having derived the mode structure of the perturbed, one-dimensional, coated, conducting sheet, we used our equations to simulate the effect of such a perturbation. We predict losses up to total attenuation depending on the exact geometry and frequency used. Independent quantitative verification of our results using a finite element solver is established. Furthermore, qualitative agreement with measurement is reported.

### (a) HFSS model

The HFSS model consisted of a solution domain (*x*,*y*,*z*; see figure 1) of dimensions 20 × 7 × 15 cm at 10 GHz and 15 × 3.5 × 15 cm at 15 GHz. The extension of the domain in the *x*-direction was chosen to be much larger than the wavelength to approximate the infinite model. Excitation in the *x*–*y*-planes was achieved with perfectly matched wave ports with 100% excitation efficiency. The conductor was modelled as a perfect electrical conductor boundary and all other boundaries were implemented as radiation boundaries. A dielectric box was implemented as the insulating sheet above the conductor. The adaptive meshing algorithm of HFSS was used to create the solution mesh with the convergence criterion Δ*S* < 0.03. It was verified that the height in the *y*-direction allowed for propagation losses without perturbation lower than 1%. The perturbing dielectric was centred in the *x*-direction touching the radiation boundaries in the *y*–*z*-planes and the *x*–*z*-plane but not in contact with the wave ports.

## Data accessibility

The experimental data, simulation data and source code may be obtained from: https://doi.org/10.5061/dryad.sbcc2fr2r [14].

## Appendix A. Bound modes of perturbed guide

The continuous fields of a bound TM mode of the perturbed guide are, omitting a factor exp (*j*(*ωt* − *βz*)), in general given by

A 1 $$\begin{array}{rl} E_{z} & =\left\{ \begin{array}{ll} E_{1}\sin(h_{1}y) & 0\leq y\leq y_{1},\\ E_{2}\cos(h_{2}y+\phi ) & y_{1}\leq y\leq y_{2},\\ E_{3}{\text{e}}^{-\gamma y} & y_{2}\leq y, \end{array}\right. \end{array}$$

A 2 $$\begin{array}{rl} H_{x} & =\left\{ \begin{array}{ll} E_{1}\frac{j\omega \epsilon_{1}}{h_{1}}\cos(h_{1}y) & y\leq y_{1},\\ -E_{2}\frac{j\omega \epsilon_{2}}{h_{2}}\sin(h_{2}y+\phi ) & y_{1}\leq y\leq y_{2},\\ E_{3}\frac{j\omega \epsilon_{3}}{\gamma }{\text{e}}^{-\gamma y} & y_{2}\leq y, \end{array}\right. \end{array}$$

A 3 $$\begin{array}{rl} E_{y} & =\left\{ \begin{array}{ll} -E_{1}\frac{j\beta }{h_{1}}\cos(h_{1}y) & y\leq y_{1},\\ E_{2}\frac{j\beta }{h_{2}}\sin(h_{2}y+\phi ) & y_{1}\leq y\leq y_{2},\\ -E_{3}\frac{j\beta }{\gamma }{\text{e}}^{-\gamma y} & y_{2}\leq y \end{array}\right. \end{array}$$

A 4 $$\begin{array}{rl} \;\text{and}\;h_{i} & =\sqrt{\omega^{2}\epsilon_{i}\mu_{0}-\beta^{2}}\;i=1,2,3, \end{array}$$

where we have used the definitions as in §3 and we define *γ* as the imaginary part of *h*_3_. To be physical, *γ* must be non-zero and positive, so the field does not diverge as *y* goes to infinity. Thus, we have the condition *β*^2^ > *ω*^2^*ϵ*_3_*μ*_0_. Using the continuity of the fields at both boundaries, we can derive the following characteristic equation:

A 5 $$-\frac{\epsilon_{2}}{h_{2}}\tan\left(h_{2}(y_{1}-y_{2})+\tan^{-1}\left(-\frac{h_{2}\epsilon_{3}}{\epsilon_{2}\gamma }\right)\right)=\frac{\epsilon_{1}}{h_{1}}\cot(h_{1}y_{1}).$$

We now show that there is no solution to equation (A 5) in the case *ϵ*_3_ > *ϵ*_1_. Because *β* is purely real for a bound mode in a lossless guide and greater than $\omega \sqrt{\epsilon_{3}\mu_{0}}$, both *h*_1_ and *h*_2_ must be imaginary. For an imaginary *h*_1_, it is straightforward to show that the right-hand side of equation (A 5) is less than zero.

For the left-hand side, we first look at the argument of the tangent function. Because *h*_2_ is imaginary, we may write the argument as

A 6 $$-j(|h_{2}|(y_{2}-y_{1})+\tanh^{-1}\left(\frac{|h_{2}|\epsilon_{3}}{\epsilon_{2}\gamma }\right)).$$

The argument in the inverse hyperbolic tangent is always greater than 1, so we get a factor of −*j* (*π*/2) from the principal branch and a positive real part. After some simplifications one can show that the left-hand side of equation (A 5) may be written as

A 7 $$\frac{\epsilon_{2}}{|h_{2}|}\coth(|h_{2}|(y_{2}-y_{1})+ℜ(\tanh^{-1}\left(\frac{|h_{2}|\epsilon_{3}}{\epsilon_{2}\gamma }\right)))>0,$$

where ℜ symbolizes taking the real part of the function. This expression is greater than zero as the argument of the hyperbolic cotangent is purely positive and real. We have shown beforehand that the right-hand side of equation (A 5) is negative. Therefore, there are no bound modes.
